# The lack of the organic cation transporter OCT1 at the plasma membrane of tumor cells precludes a positive response to sorafenib in patients with hepatocellular carcinoma

**DOI:** 10.18632/oncotarget.15029

**Published:** 2017-02-02

**Authors:** Andreas Geier, Rocio I.R. Macias, Dominik Bettinger, Johannes Weiss, Heike Bantel, Daniel Jahn, Ruba Al-Abdulla, Jose J.G. Marin

**Affiliations:** ^1^ Division of Hepatology, Department of Medicine II, University Hospital Würzburg, Würzburg, Germany; ^2^ Experimental Hepatology and Drug Targeting, CIBERehd, IBSAL, University of Salamanca, Salamanca, Spain; ^3^ Department of Medicine II, University Hospital Freiburg, Freiburg, Germany; ^4^ Department of Gastroenterology, Hepatology and Endocrinology, Hannover Medical School, Hannover, Germany

**Keywords:** organic cation transporter, chemoresistance, hepatocellular carcinoma, tyrosine-kinase inhibitor, sorafenib

## Abstract

**Background:**

Sorafenib is the drug of choice in the treatment of advanced hepatocellular carcinoma (HCC). Beneficial effects are limited by mechanisms of chemoresistance, which include downregulation and/or impaired function of plasma membrane transporters accounting for drug uptake. The organic cation transporter 1 (OCT1) plays a major role in sorafenib uptake and decreased expression in HCC has been associated with poorer response.

**Methods:**

The multicenter retrospective TRANSFER study involved tumor biopsies from 39 patients with advanced HCC and sorafenib therapy for ≥4 wk. Endpoint was the relationship between clinicopathological features and immunohistological result. Immunostaining was performed using specific primary anti-OCT1-head and anti-OCT1-tail antibodies. Tumors were classified according to a simplified staining score as absent, weak, moderate or strong, taking into account the localization of the staining at the plasma membrane as positive or negative.

**Results:**

Results confirmed OCT1 downregulation in half of the cases investigated (10% absent, 38% weak). However, only one third of tumors expressing OCT1 displayed plasma membrane location (15% vs. 36% cytosolic expression). When comparing HCC with and without OCT1 expression, no different sorafenib response was found. When tumors expressing OCT1 at the plasma membrane were considered separately, a marked longer survival was found (Log Rank p<0.001). No association between OCT1 expression at the plasma membrane with tumor stage, previous treatment with TACE or radiological response was seen.

In conclusion, these results indicate that the presence of OCT1 at the plasma membrane, rather than its expression levels, is related to better outcome of HCC patients treated with sorafenib.

## BACKGROUND

Hepatocellular carcinoma (HCC) is the sixth most common cancer worldwide and the third cause of death due to cancer [1–4]. Over the last two decades the expected outcome of patients with HCC has been improved considerably from a dismal prognosis to 30–40% of patients in developed countries nowadays being diagnosed at early stages allowing for curative treatment approaches such as local ablation, tumor resection or liver transplantation [5, 6]. However, the prognosis of advanced tumors has not changed considerably despite the introduction of targeted systemic treatment. Since 2008 the multikinase inhibitor sorafenib has become the standard of systemic therapy for advanced stage HCC and its approval represents a breakthrough in the management of advanced tumors [7]. Sorafenib treatment improved the time to progression (TTP) and extended overall survival by 2.8 and 2.3 months compared to placebo in advanced HCC patients (10.7 months vs 7.9 months in SHARP; 6.5 months vs 4.2 months in Asia-Pacific) [7, 8]. Data from second line treatment indicated a statistically significant difference in outcome between MET-high populations treated with placebo and tivantinib (median overall survival of 7.2 months for tivantinib compared with 3.8 months for placebo) whereas no such difference could be observed in MET-low populations [9]. These data support the general need for a personalized strategy in the treatment of HCC according to the presence of molecular targets in each tumor [10]. Thus, antitumoral effects of sorafenib are heterogeneous in different patients, which makes necessary to identify biomarkers either in tumor or peripheral blood to predict patient outcomes in a personalized manner. On one hand, blood biomarkers such as phorbol myristate acetate-induced phosphorylation of extracellular signal–related kinase (ERK) have been identified in peripheral blood lymphocytes [11]. Furthermore, baseline pERK expression was identified as a promising intratumoral marker of response since HCC patients whose tumors expressed higher levels of this target structure had a longer TTP following treatment with sorafenib in the phase II study [12]. Besides the preservation of the molecular targets of this drug in tumor cells, the absence of respective drug transporters also represents a putative predictor of poor response to the treatment. The mechanism of action of sorafenib depends on its access to the intracellular site of action on transmembrane tyrosine kinase receptors, which may be affected by changes in the expression and activity of transporters accounting for its uptake. The organic cation transporter-1 (OCT1, gene symbol *SLC22A1*) has been suggested to play a major role in this process [13, 14]. OCT1 functions as an electrogenic, sodium- and proton-independent bidirectional polyspecific transporter [15]. Human OCT1 is located at the basolateral membrane of hepatocytes, enterocytes, and renal proximal tubular cells, where it mediates the facilitated transport of a variety of structurally diverse organic cations, including endogenous and xenobiotic compounds, such as toxins and drugs [16]. Its role in sorafenib uptake has prompted us and other groups to investigate the usefulness of determining OCT1 expression in tumor tissue as a prognostic biomarker for the response to systemic treatment of HCC with this drug [13, 14, 17]. The identification of polymorphic genetic variants of human OCT1 that severely affect transport activity suggested that some of the inter-individual differences in response to cationic drugs may be caused by variable activity of this transporter among tumors [14]. Recently, two novel *SLC22A1* variants R61S fs*10 and C88A fs*16 encoding truncated proteins unable to reach the plasma membrane of liver tumor cells together with an abundant proportion of aberrant alternative splicing have been described as common features in HCC [13]. In the present study we have addressed the question on whether the presence of the transporter at the plasma membrane, rather than overall OCT1 expression (mRNA/protein) levels in tumor cells, is a better prognostic marker for the outcome of HCC patients treated with sorafenib.

## RESULTS

### Patient characteristics

Patients in the *TRANSFER* study were 67.4±1.6 years of age and predominantly male. Most frequent underlying chronic liver diseases were alcoholic and viral hepatitis (Table 1). Child-Pugh score (CPS) at the time of sorafenib initiation was CPS A in two thirds of the patients while a minority was either CPS B or could not be calculated due to missing laboratory values. HCC diagnosis was invariably based on liver histology with an equal distribution of BCLC stages B and C (38.5% and 41.0%, respectively). 87.2% of the patients underwent surgical resection or locoregional treatment, either transarterial chemoembolization (TACE) or radiofrequency ablation, or both prior to sorafenib treatment.

**Table 1:** Clinical information on patients and tumors

*Patients*		
Age (mean±EEM)	67.4±1.6	
Age range	49-87	
Male	35	(89.7%)
Female	4	(10.3%)
***Sorafenib pretreatment***		
No	5	(12.8%)
Resection	7	(18.0%)
TACE	12	(30.8%)
RFA	1	(2.6%)
Resection, TACE	10	(25.6%)
TACE, RFA	2	(5.1%)
Resection, TACE, RFA	2	(5.1%)
***HCC Etiology***		
Alcohol use	11	(28.2%)
Hepatitis B	4	(10.3%)
Hepatitis C	11	(28.2%)
Hemochromatosis	2	(5.1%)
NAFLD	3	(7.7%)
Unknown	8	(20.5)
***Child-Pugh status***		
Child-Pugh A	26	(66.6%)
Child-Pugh B	7	(18.0%)
Unknown	6	(15.7%)
***BCLC classification***		
A	1	(2.5%)
B	15	(38.5%)
C	16	(41.0%)
Unknown	7	(18.0%)

Patients (n=39) were diagnosed of suffering from hepatocellular carcinoma and were included in the study based on eligibility criteria described in detail in Method section.

### Immunohistochemical staining of OCT1

**Figure 1:**
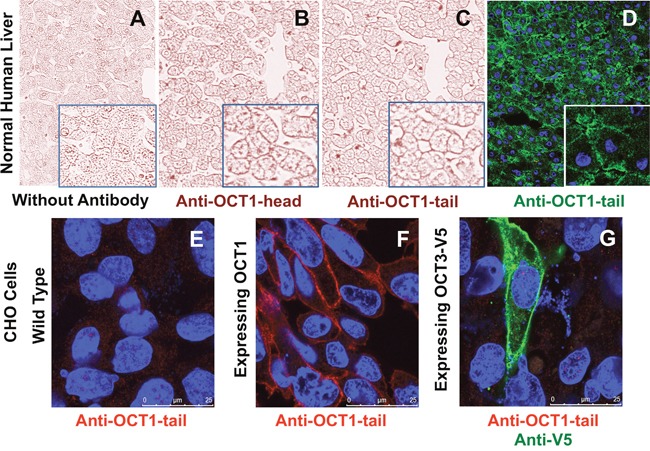
Representative images of immunohistochemical analysis of OCT1 in healthy human liver using no primary antibody (negative control) **A**. or two different primary rabbit polyclonal anti-OCT1 antibodies LS-C31870 **B**. and LS-C161155 **C**. raised against the head and tail of the protein, respectively. Immunofluorescence combined with confocal microscopy of normal liver tissue stained with anti-OCT1-tail antibody **D**. Study of the selectivity of anti-OCT1-tail antibody using Chinese hamster ovary (CHO) cells either wild type **E**. or expressing human OCT1 **F**. or OCT3 tagged with V5 antigen **G**. The nuclei were stained with Dapi.

Prior to analyze the presence of OCT1 at the plasma membrane in HCC samples collected from these patients, two different antibodies raised against different regions of the protein were tested on healthy liver tissue (Figure 1A–1D). In the negative control, i.e., when neither of these two antibodies was used before incubation with the secondary antibody, no signal was detected (Figure 1A). In contrast, both LS-C31870 anti-OCT1-head (Figure 1B) and LS-C161155 anti-OCT1-tail (Figure 1C) antibodies were able to detect OCT1 at the hepatocyte plasma membrane in immunohistochemical analyses. Similar results were obtained in immunofluorescence analysis using anti-OCT1-tail antibody (Figure 1D). In contrast, strong noise and poor specific signal was obtained with the anti-OCT1-head antibody (data not shown), which precludes its use in this technique.

To evaluate the specificity of the OCT1 signal detected at the plasma membrane of human liver cells, *in vitro* experiments were carried out using CHO cells. The immunofluorescence assays showed the absence of anti-OCT1-tail antibody reactivity with endogenous proteins of these hamster cells (Figure 1E). In contrast, when CHO cells were transduced with human OCT1, immunofluorescence analysis using the anti-OCT1-tail antibody permitted to detect this protein at the plasma membrane (Figure 1F). No signal was found when CHO cells were transduced with human OCT3-V5, even if they expressed the recombinant protein at the plasma membrane, as detected using an anti-V5 antibody (Figure 1G). These results demonstrated that the anti-OCT1-tail antibody used in the immunohistochemical analysis of OCT1 in patient samples has no cross-reactivity with OCT3.

### Localization and quantification of OCT1 protein staining in HCC tumor tissue

**Figure 2:**
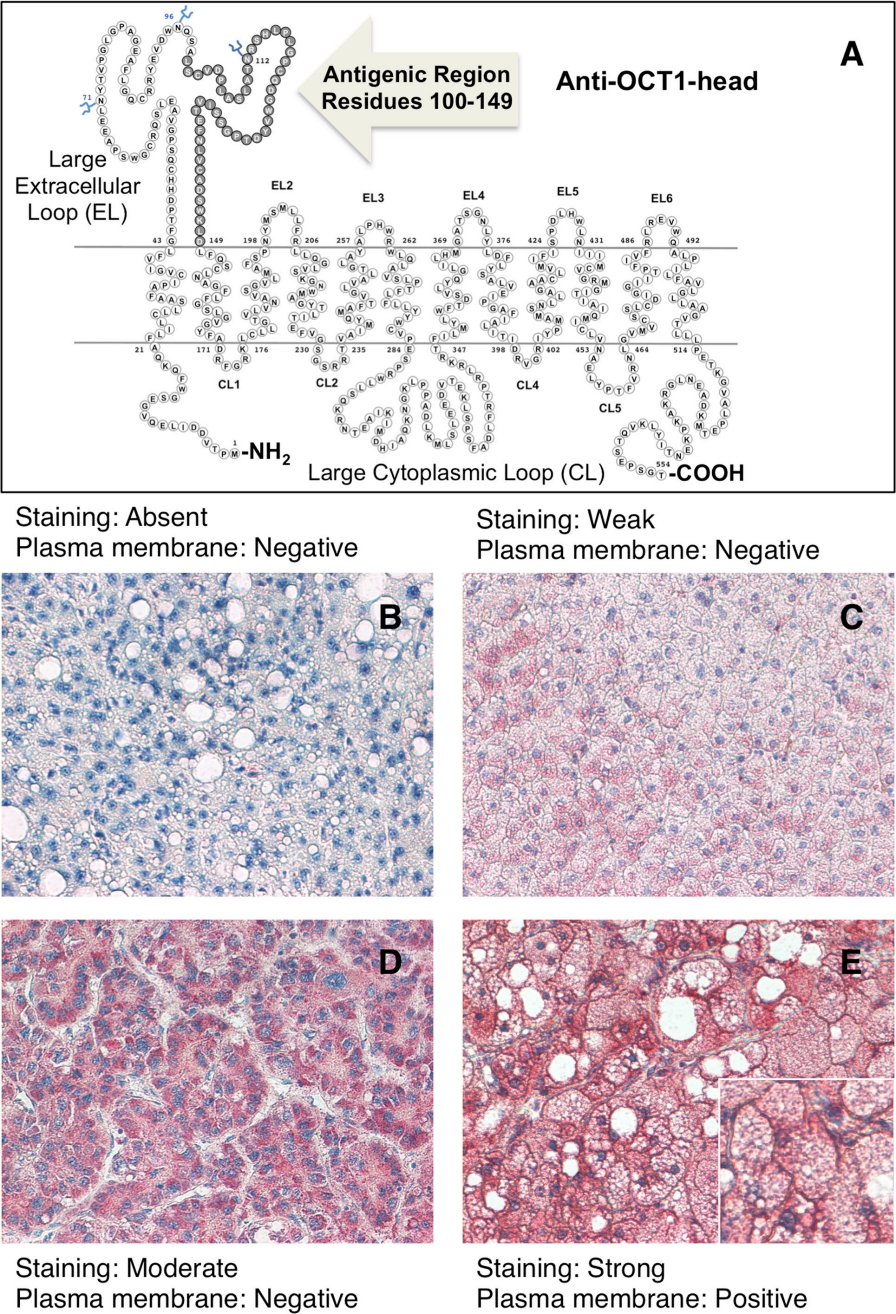
Schematic drawing of OCT1 showing the antigenic region used to raise the rabbit polyclonal LS-C31870 anti-OCT1-head antibody **A**. Representative images of immunohistochemical analysis showing the criteria used for hepatocellular carcinoma score depending upon the degree of slides staining with anti-OCT1-head antibody as: absent **B**. weak **C**. moderate **D**. and strong **E**. and the lack (B, C, D) or the presence **E**. of staining at the plasma membrane.

**Figure 3:**
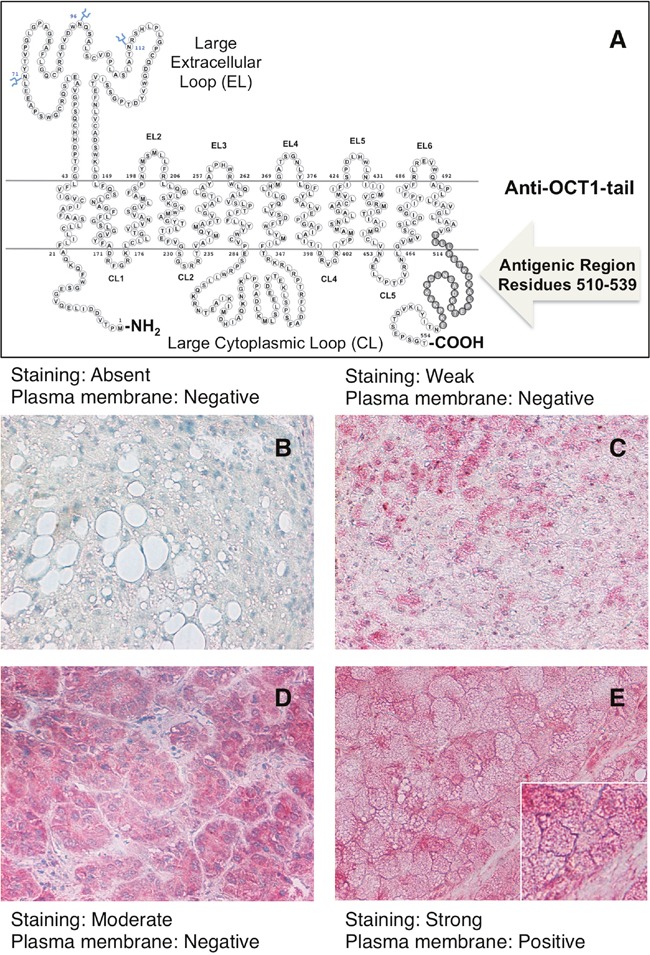
Schematic drawing of OCT1 showing the antigenic region used to raise the rabbit polyclonal LS-C161155 anti-OCT1-tail antibody **A**. Representative images of immunohistochemical analysis showing the criteria used for hepatocellular carcinoma score depending upon the degree of slides staining with anti-OCT1-tail antibody as: absent **B**. weak **C**. moderate **D**. and strong **E**. and the lack (B, C, D) or the presence **E**. of staining at the plasma membrane.

**Figure 4:**
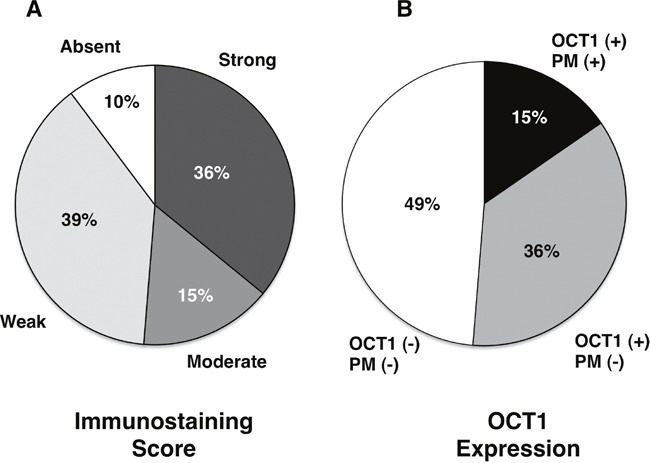
Classification of patients with hepatocellular carcinoma based only on the degree of OCT1 staining with anti-OCT1-head or anti-OCT1-tail antibodies **A**. or taking also into account the presence of the staining at the plasma membrane **B**.

Due to frequent nonsense mutations and aberrant splicing, an important proportion of OCT1 mRNA found in HCC is expected to generate non-functional truncated peptides [13]. Thus, to analyze the presence of OCT1 in patient samples we have used two antibodies raised against the N-terminal region, i.e., the head of the protein (Figure 2A) and the C-terminal region, i.e., the OCT1-tail (Figure 3A). Regarding the ability to detect the presence of OCT1 at the plasma membrane no difference was observed between both antibodies in all samples analyzed, probably because most protein found at the plasma membrane contained the complete sequence. To make easier the interpretation of the results, a simplified staining score of quantitative OCT1 protein expression in HCC cells was established (see Materials and Methods section). Localization of the OCT1 staining at the plasma membrane was classified as either positive or negative (Figure 2B–2E and Figure 3B–3E). In spite of marked interindividual variability, ranging from absent to strong (Figure 2B–2E and Figure 3B–3E), the results from the immunohistochemical analysis have confirmed previous observations on the downregulation of OCT1 in HCC when measured as the abundance of OCT1 mRNA or protein [13, 17, 19–21]. Indeed, when the tumors were classified according to the intensity of staining approximately half of them displayed marked (defined as moderate or strong staining scores) OCT1 expression (Figure 4A). Because an important part of OCT1 in HCC cells corresponds to truncated peptides [13], it was not surprising that staining at the plasma membrane was detected in only one third of HCC samples that markedly expressed OCT1 (Figure 4B). Interestingly, the intracellular signal obtained with the anti-OCT1-tail antibody was somehow weaker than that obtained with the anti-OCT1-head antibody. Since the use of either antibody gave the same results in the global analysis carried out here, from now on we will describe and discuss the results without distinguishing between anti-OCT1-head and –tail antibodies.

### Relationship between treatment response and OCT1 abundance/localization

**Figure 5:**
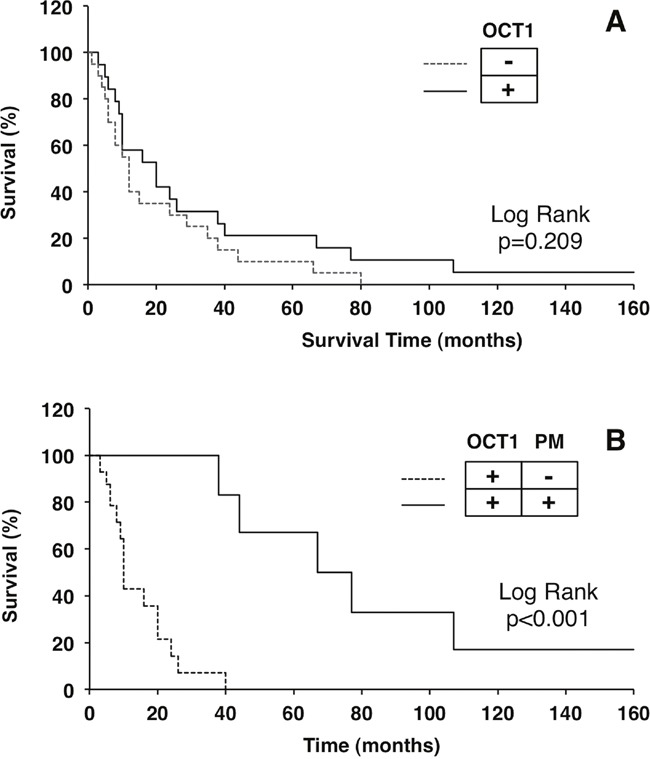
Kaplan-Meier plots of survival after starting hepatocellular treatment with sorafenib Patients were classified based only on the degree of OCT1 staining. **A**. or taking also into account the presence of the staining at the plasma membrane. **B**. Comparisons were performed with the Log Rank (Mantel-Cox) test.

**Figure 6:**
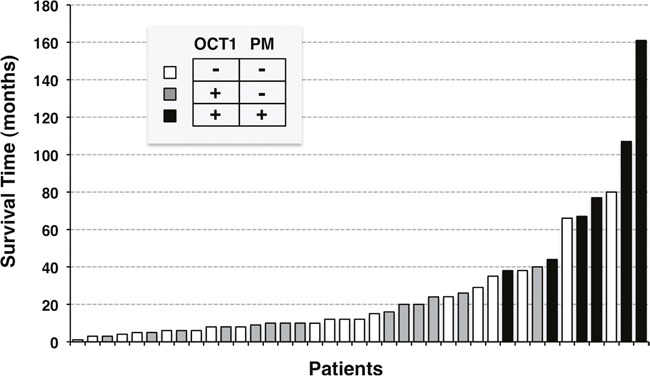
Waterfall plot of the clinical response to sorafenib treatment as determined by overall survival after starting systemic pharmacological treatment with sorafenib Patients were classified based on the degree of OCT1 staining and the presence of the staining at the plasma membrane.

**Figure 7:**
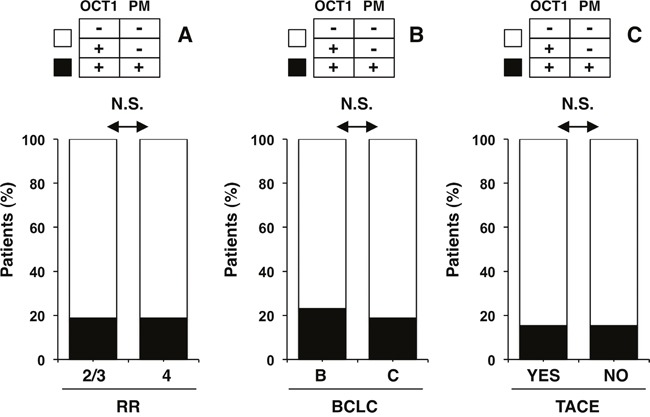
Absence of relationship between the expression of OCT1 and its presence at the plasma membrane with commonly used clinical criteria, such as **A**. radiological response (RR), whose code was: 1) Complete response; 2) Partial response; 3) Stable disease; 4) Progression disease; **B**. BCLC stage B versus C; **C**. pretreatment by TACE. N.S., p>0.05 by the Fisher's exact test.

To assess whether these findings are relevant from the functional point of view and to understand the role of OCT1 transport function in the sensitivity to sorafenib we next analyzed the treatment response according to OCT1 abundance and localization. This was consistent with results obtained using Kaplan-Meier plots to study the survival of patients treated with sorafenib. The general comparison of patients bearing HCC with and without OCT1 protein expression revealed no significant difference in survival (Figure 5A). Because the absence of the transporter at the plasma membrane precludes the function of the protein as a transporter, even if it is highly expressed in tumor cells, we further analyzed the role of subcellular localization. When patients with tumors with evident expression of OCT1 at the plasma membrane were segregated into a different group, a markedly longer survival was observed in patients with positive membrane staining compared to those with a negative value for this criterium (Figure 5B). Waterfall plotting illustrates a more pronounced beneficial effect of sorafenib treatment in patients with expression of OCT1 at the plasma membrane (Figure 6). Analysis of individual cases reveals that all six patients with positive membrane staining were among the top ten survivors in this study. The effect of positive OCT1 staining at the plasma membrane on radiological response was less pronounced and did not reach statistical significance (Figure 7A). This may be accounted for by the retrospective nature of this study, which lacks standardized staging algorithms.

### Relationship between OCT1 expression and tumor stage or TACE pretreatment

Adaptive changes in target molecule expression during tumor progression and after pretreatment have been described in the past. Therefore, we analyzed the impact of tumor stage and previous TACE (total of 26 patients) on OCT1 staining at the plasma membrane. Notably, when we investigated tumor stage according to BCLC criteria (Figure 7B) and pretreatment with TACE (Figure 7C), no relationship of these conditions with the presence of OCT1 at the plasma membrane of HCC cell was observed.

## DISCUSSION

OCT1 plays a major role in the hepatocellular uptake of sorafenib, the so far only licensed systemic treatment for HCC [7]. It has been recently shown that overall OCT1 (mRNA/protein) levels in tumor tissue detected using RT-QPCR, mRNA-microarray or immunoblotting may serve as a prognostic biomarker for the response to systemic treatment of HCC with this drug [13, 14, 17]. In the present study, we have taken a step forward by addressing the question on whether the presence of the transporter at its functional site in the plasma membrane, rather than its overall expression in tumor cells, is a better prognostic marker for the outcome of HCC patients treated with sorafenib. As the principal finding of the *TRANSFER* study, we here show for the first time that the site-specific absence of the transporter at the plasma membrane precludes a favorable overall survival, even if OCT1 is highly expressed in the cytosol of tumor cells. It is important to highlight that intracellular OCT1 is not expected to contribute to sorafenib uptake and hence it does not play a role in allowing the drug reaching its intracellular molecular targets.

Marked interindividual variability regarding OCT1 mRNA and/or protein in normal liver has been reported and the underlying genetic and non-genetic factors have been partly elucidated [22]. Whether similar mechanisms are involved in the downregulation of OCT1 found in HCC [13, 23] is unknown. Some of the causes for this decreased expression could be epigenetic, thus DNA methylation of *SLC22A1* gene has been associated with downregulation of OCT1 in HCC [23], but complete elucidation of the low expression of this transporter in tumor cells is currently missing. The clinical repercussion of the low or absent expression of OCT1 in HCC has been reported to include impaired drug uptake and presumably a reduced clinical effect of sorafenib [13]. Subsequent studies have supported this concept by reporting a relationship between intratumoral levels of OCT1 mRNA and the response to sorafenib [17]. However, it should be considered that non-functional aberrant variants constitute a marked proportion of synthesized OCT1 mRNA. Under these circumstances, it seems that the detection of OCT1 at the protein level would better reflect OCT1 function. Using this approach OCT1 downregulation in HCC has been also confirmed [21]. The main contribution of the present study is to highlight that, in addition to reduced OCT1 expression (mRNA/protein) levels in the tumor, the reduction in functional OCT1 at the plasma membrane of tumor cells plays a key role among the multifactorial mechanisms of chemoresistance (MOC) determining the response of HCC to sorafenib. It is interesting to note that neither the stage of the tumor nor the pretreatment with TACE affected the presence of OCT1 at the plasma membrane. Moreover, no relationship with the radiological response was found. This indicates that a more efficient sorafenib uptake through OCT1 results in a long-term beneficial effect, which was not seen at the moment when the unscheduled radiological follow-up of these patients had been carried out in this retrospective study.

It is also important to mention that although pharmacological activity of sorafenib is dependent on its intracellular concentration, sensitivity to this drug can be reduced by several mechanisms of chemoresistance (MOCs) other than reduced uptake through OCT1 [24]. Thus, *in vitro* induction of chemoresistance in human hepatoma cells by continuous exposure to sorafenib results in HCC cell clones with marked intrinsic differences regarding their MOC. In spite of maintained OCT1 expression, some clones developed chemoresistance to sorafenib by enhanced efflux through upregulation of the ABC protein, MRP3 [25] or activation of RAF kinases and PI3K/AKT pathway [26]. A role in MOC related to changes in the expression of ABC pumps, mainly ABCG2, has also been reported in clinical samples of HCC [21].

The fact that sorafenib was still moderately effective in patients bearing tumors with negligible expression of OCT1 is consistent with the fact that, although OCT3 (*SLC22A3*) expression in normal liver tissue is markedly lower than that of OCT1, OCT3 might replace OCT1 as major organic cation transporter in some cases of HCC. Analysis of mRNA levels for OCT1 and OCT3 in paired samples of HCC and surrounding liver tissue revealed downregulation of both transporters in most cases. However, in some tumors with dramatic downregulation of OCT1, the expression of OCT3 was preserved or increased [20, 23]. Consequently, in these tumors, OCT3 could be able to mediate enough sorafenib uptake to reach effective intracellular concentrations.

Of note, when examined in patients with HCC at earlier stages who have underwent liver resection or transplantation, OCT1 downregulation correlated with tumor progression [20]. However, in our series of patients with advanced HCC this relationship was no longer evident. The proportion of HCC with negative OCT1 immunostaining was similar in tumors of BCLC grade B and C.

In conclusion, these results indicate that the presence at the plasma membrane, rather than the overall OCT1 expression, is related with a favorable outcome in HCC patients treated with sorafenib. Although the present study has been focused on the clinical response to sorafenib, the interesting results obtained here suggest that further investigations are required to elucidate whether similar relationship is also valid for other TKIs, such as novel and promising MET inhibitors tivantinib and cabozantinib, currently under clinical evaluation for the treatment of HCC [9]. A prospective study is warranted to investigate the use of OCT1 immunostaining for the guidance of systemic first line treatment with sorafenib in the future.

## MATERIALS AND METHODS

### Study population and eligibility

The *TRANSFER* (TRANsporter SoraFEnib Response) study was a multicenter retrospective investigation involving liver tumor biopsy samples collected for diagnostic or treatment purposes of HCC in patients who were treated with sorafenib in three German centers: University Hospital Würzburg (n=14), University Hospital Freiburg (n=20) and Hannover Medical School (n=5) from 2007 to 2015. Clinical data including hepatorenal function (MELD), BCLC stage, previous locoregional therapy, radiological response and survival were recorded. The study was approved by the Institutional Review Board of each participating center and conducted according to the principles expressed in the Declaration of Helsinki. Written informed consent for the use of patient tissue samples was waived and clinical data were anonymized. Clinical and tumor characteristics are shown in Table 1.

Inclusion criteria were: i) Diagnosis of HCC based on pathology or imaging techniques obtained by dynamic contrast-enhanced multidetector CT scan or MRI according to the EASL guidelines [18]; ii) Sorafenib therapy for advanced HCC with known outcome (survival, radiological response), minimum duration of 4 weeks; iii) Compensated liver function prior to therapy (Child Pugh Class A or B); iv) Good Performance Status (PS 0-2); v) Availability of formalin fixed tumor tissue for histological analysis; vi) Time interval between tissue acquisition and start of sorafenib treatment no longer than 18 months. In fact, the mean time interval was 13.6 ± 22.2 months (median 7.1 months) including six patients with extended time interval who were accepted for inclusion by the lead investigator.

Exclusion criteria were: i) “Mixed” tumors as diagnosed by histological analysis; ii) No definite diagnosis of HCC; iii) Systemic chemotherapy, other than sorafenib, between tissue acquisition and start of sorafenib treatment. However, local therapy such as transarterial chemoembolization was allowed; iv) Decompensation of liver function (Child-Pugh Class C) before initiation of sorafenib treatment; v) Performance Status PS>2.

### Immunohistochemistry analysis

Formalin-fixed, paraffin embedded HCC tissue was used. Immunostaining was performed on whole sections from paraffin-embedded material using two different primary anti-OCT1 rabbit polyclonal antibodies obtained from LifeSpan BioSciences, Madrid, LS-C31870 and LS-C161155, raised against the N-terminal (residues 100-149) and C-terminal (residues 510-539) regions of human OCT1 protein, respectively. Mouse and rabbit AP/Fast Red (ABC) Detection IHC Kit (Abcam) was used as briefly described below. After deparaffinization in xylene and rehydration in a graded series of ethanol, samples were subjected to antigen retrieval at pH 6.0 using steamer heating for 20 min, washed four times in buffer (pH 7.4), incubated with protein blocking solution for 5 min to block nonspecific background staining and incubated 30 min with one of the two anti-OCT1 antibodies used here (both diluted 1/100), followed by 15 min with biotinylated secondary antibody against the primary antibody, 15 min with streptavidin alkaline phosphatase, and 10 min with the substrate-chromogen fast red dissolved in naphthol phosphate buffer. After each of the previous step samples were washed four times in buffer. All procedures were carried out at room temperature unless otherwise specified. Slides were counter-stained with hematoxylin and mounted with aqueous mounting medium. In negative control sections, wherein primary antibody was omitted, no immunostaining was observed (Figure 1A).

The slides were visualized under a light microscope and immunohistochemical stainings were reviewed independently by two observers, who were blinded to clinical data. Tumors were classified according to a simplified staining score as absent, weak, moderate or strong, and taking into account the localization of the staining at the plasma membrane as positive or negative (Figure 2B–2E and Figure 3B–3E). Slides with discrepancies were visualized a second time by the two observers together to achieve a consensus.

### OCT1 and OCT3 *in vitro* expression

Using total RNA isolated from healthy liver, the open reading frames (ORF) of human OCT1 and OCT3 were amplified by reverse transcription followed by high-fidelity PCR using AccuPrime Pfx DNA polymerase (Invitrogen, Thermo Fisher Scientific, Madrid, Spain). The purified amplicon was cloned into a modified pWPI lentiviral vector, which was manipulated to include the V5 antigen as a tag linked at the C-terminal of the transporter protein. Recombinant lentiviruses were produced in HEK293T cells transfected using a standard polyethylenimine (PEI) protocol with the transfer vectors pWPI-OCT1/pWPI-OCT3, encoding both the desired OCT1/OCT3 and EGFP, and the packaging plasmids psPAX2 and pMD2.G. Viral titers were determined by infection of HEK293T cells with serial dilutions of the viral solution, and analysis of EGFP-positive cells was carried out with a FACSCalibur flow cytometer (BD Biosciences, Madrid). These lentiviral vectors were used to transduce Chinese hamster ovary (CHO) cells 48 h before immunofluorescence analyses were carried out.

### Immunofluorescence analysis

Human liver cryosections (5 μm thin) or cultured CHO cells were fixed using ice-cold methanol for 3 min. After blocking with PBS supplemented with 5% fetal bovine serum for 30 min, wild type cells or those expressing OCT1 or OCT3-V5 were incubated for 1 h with primary antibodies against OCT1-head or OCT1-tail and/or against V5 (mouse monoclonal anti-V5, R96025, Invitrogen). As secondary antibodies anti-mouse or anti-rabbit Alexa 594- or Alexa 488-conjugated antibodies, both from Life Technology, were used as appropriate. The nuclei were stained with Dapi. The images were obtained using a confocal microscope (Leica TCS SP2).

### Statistical analysis

The statistical analysis has been performed using SPSS (IBM® SPSS® Version 20.0 for Mac). Fisher's exact or log-rank (Mantel-Cox) tests were used as appropriate.
